# Endosymbiosis allows *Sitophilus oryzae* to persist in dry conditions

**DOI:** 10.3389/fmicb.2023.1199370

**Published:** 2023-07-11

**Authors:** Sthandiwe Nomthandazo Kanyile, Tobias Engl, Abdelaziz Heddi, Martin Kaltenpoth

**Affiliations:** ^1^Department of Insect Symbiosis, Max Planck Institute for Chemical Ecology, Jena, Germany; ^2^INSA Lyon, Université de Lyon, Villeurbanne, France

**Keywords:** cuticle, desiccation stress, rice-weevil, symbiosis, tyrosine

## Abstract

Insects frequently associate with intracellular microbial symbionts (endosymbionts) that enhance their ability to cope with challenging environmental conditions. Endosymbioses with cuticle-enhancing microbes have been reported in several beetle families. However, the ecological relevance of these associations has seldom been demonstrated, particularly in the context of dry environments where high cuticle quality can reduce water loss. Thus, we investigated how cuticle-enhancing symbionts of the rice-weevil, *Sitophilus oryzae* contribute to desiccation resistance. We exposed symbiotic and symbiont-free (aposymbiotic) beetles to long-term stressful (47% RH) or relaxed (60% RH) humidity conditions and measured population growth. We found that symbiont presence benefits host fitness especially under dry conditions, enabling symbiotic beetles to increase their population size by over 33-fold within 3 months, while aposymbiotic beetles fail to increase in numbers beyond the starting population in the same conditions. To understand the mechanisms underlying this drastic effect, we compared beetle size and body water content and found that endosymbionts confer bigger body size and higher body water content. While chemical analyses revealed no significant differences in composition and quantity of cuticular hydrocarbons after long-term exposure to desiccation stress, symbiotic beetles lost water at a proportionally slower rate than did their aposymbiotic counterparts. We posit that the desiccation resistance and higher fitness observed in symbiotic beetles under dry conditions is due to their symbiont-enhanced thicker cuticle, which provides protection against cuticular transpiration. Thus, we demonstrate that the cuticle enhancing symbiosis of *Sitophilus oryzae* confers a fitness benefit under drought stress, an ecologically relevant condition for grain pest beetles. This benefit likely extends to many other systems where symbiont-mediated cuticle synthesis has been identified, including taxa spanning beetles and ants that occupy different ecological niches.

## Introduction

The threat of desiccation remains one of the most significant challenges that terrestrial animals face. This threat is exacerbated in insects because of their small size and large surface area to volume ratio, which makes them particularly prone to rapid water loss (Bazinet et al., 2010; Bujan et al., 2016). Despite this, insects show a remarkable ability to maintain water balance using an arsenal of behavioral, morphological and physiological adaptations (Chown et al., 2011). These adaptations involve mechanisms to acquire water, and to reduce or tolerate water loss, thereby enabling insects to subsist in dry and hot environments (Sheikh et al., 2017; Fanning et al., 2019).

Adaptations to low humidity and high temperature often overlap, because in both conditions, the risk of dehydration must be mitigated by water conserving mechanisms. Insects can employ behavioral adaptations to limit water loss. For instance, workers of thermophilic ant genera that inhabit arid or semi-arid regions frequently retreat to “thermal refuge pockets” in shadows or slightly elevated points to avoid overheating (Perez and Aron, 2020). Morphological adaptations such as increased body size, leg length and body hairs have also been shown to contribute significantly to body cooling (Sommer and Wehner, 2012; Willot et al., 2016; Perez and Aron, 2020). Physiological mechanisms such as the storage of high amounts of initial body water content may also provide alternative strategies for dealing with thermal and dry stress. This has been demonstrated in *Drosophila melanogaster*, where desiccation resistance correlated with decreased water loss and higher amounts of stored water, mediated by increased glycogen stores (Bazinet et al., 2010). Furthermore, insects can improve the physical and chemical properties of the primary portal of water loss, i.e., the insect cuticle, to reduce trans-cuticular evaporation (Bazinet et al., 2010; Stinziano et al., 2015). While insects can lose water during oviposition and excretion of feces, a substantial amount is continuously lost through transpiration from respiratory and cuticular surfaces. Concordantly, trans-cuticular water loss has been shown to account for up to 80% of an insect’s water budget (Quinlan and Gibbs, 2006).

As skin and exoskeleton, the cuticle covers the entire surface of an insect, and its structural integrity plays a critical role in limiting water loss (Moussian, 2013). The insect cuticle is a heterogeneous structure that is composed of several layers. The outermost layer (epicuticle) is a hydrophobic lipid layer mainly consisting of hydrocarbons, the quantity and composition of which can be rapidly adjusted by the insect in response to changes in temperature and humidity (Howard and Blomquist, 2005; Bazinet et al., 2010; Menzel et al., 2017). Below the epicuticle lies the exocuticle, which is a matrix of cross-linked proteins and chitin (Andersen, 2010; Noh et al., 2016; Evison et al., 2017). Importantly, the exocuticle can be modified through the processes of sclerotization and melanization which involve the incorporation of phenolic compounds and pigments, respectively, leading to increased hardness and darkness of the cuticle (Andersen, 2010; Noh et al., 2016; Evison et al., 2017). Thus, the incorporation of these phenolic compounds serves to increase the rigidity, thickness, density and hydrophobicity of the cuticle, limiting transpiration and enhancing desiccation resistance. Indeed, several studies have demonstrated that desiccation resistance is often accompanied by changes in cuticular permeability and that increased melanization correlates with reduced rates of water loss (Gibbs et al., 2004; Stinziano et al., 2015; Farnesi et al., 2017; Ajayi et al., 2020).

Indispensable to the process of sclerotization and melanization is the semi-essential aromatic amino acid tyrosine, because it serves as the precursor for melanin and the phenolic compounds used for sclerotization (Andersen, 2010; Noh et al., 2016; Evison et al., 2017). However, insects lack the shikimate pathway required for the synthesis of aromatic molecules such as tyrosine (Evison et al., 2017). The demand for tyrosine can be met via dietary sources, especially when insects feed on proteinaceous diets (Kramer and Hopkins, 1987). However, many herbivorous insects feed on nutritionally limiting diets and they may benefit from symbiotic associations with microbes that provide precursors for tyrosine biosynthesis. This is particularly the case in beetles, where high tyrosine investment is required for the formation of their strongly sclerotized cuticle and elytra. Tyrosine provisioning symbioses have now been identified in several different beetle taxa (Kuriwada et al., 2010; Oakeson et al., 2014; Vigneron et al., 2014; Anbutsu et al., 2017; Hirota et al., 2017; Engl et al., 2018; Kiefer et al., 2021, 2022). Additionally, this phenomenon has also been observed in some ant genera (Klein et al., 2016; Sinotte et al., 2018; Jackson et al., 2021). This suggests that symbiotic associations may be a widespread means of solving the problem of tyrosine limitation and the resulting deficiencies in cuticular traits. Thus, tyrosine-provisioning endosymbionts may allow insects to cope with challenging environments, such as those characterized by dryness, where high cuticle quality may be particularly beneficial.

One particularly dry niche that is colonized by insects is that of grain storage facilities, where low ambient humidity is maintained to prevent the growth of mould fungi (Hernandez Nopsa et al., 2015). Additionally, the grain itself may be rich in starch and carbohydrates but limited in nitrogenous compounds (Šramková et al., 2009). Concordantly, out of the over 150 species of insects listed as grain pests, only 10–15 species occur frequently, and most of these are beetles (White et al., 2011; Jian and Jayas, 2012). Some of these grain pest beetles have been demonstrated to be in mutualistic association with microbes that supply them with precursors for cuticle formation, i.e., *Rhyzopertha dominica* (Bostrichidae, Kiefer et al., 2022), *Sitophilus spp.* (Curculionidae, Oakeson et al., 2014; Vigneron et al., 2014), and *Oryzaephilus surinamensis* (Silvanidae, Engl et al., 2018). In the latter two taxa, it has been shown that the experimental removal of symbionts results in perturbations in cuticle biosynthesis, with artificially symbiont-depleted (aposymbiotic) beetles exhibiting thinner and less melanized cuticles (Vigneron et al., 2014; Engl et al., 2018). Furthermore, the enhanced cuticle plays a vital role in protecting symbiotic beetles from desiccation stress, predators and pathogens (Engl et al., 2018; Kanyile et al., 2022).

The rice weevil *S. oryzae* is a grain pest beetle that is associated with the intracellular bacterial symbiont *Sodalis pierantonius* (Vigneron et al., 2014; Maire et al., 2020). Specifically, *S. pierantonius endo*symbionts supplement the weevil diet with constituents that are poorly represented in wheat grains, such as biotin, riboflavin, pantothenic acid, pyridoxine, folic acid, essential amino acids and aromatic amino acids, particularly phenylalanine and tyrosine (Vigneron et al., 2014). Symbiont elimination and nutrient supplementation experiments have demonstrated that endosymbionts increase fecundity, reduce larval developmental time, and improve flight ability of weevils (Grenier et al., 1994; Heddi et al., 1999; Carvalho et al., 2014). Similarly to *O. surinamensis*, *S. oryzae* endosymbionts contribute to cuticle biosynthesis through their provisioning of aromatic amino acids, which not only accelerates cuticle development but also ultimately results in symbiotic beetles exhibiting thicker and more melanized cuticles (Vigneron et al., 2014). Given the widespread occurrence of tyrosine-supplementing symbioses in beetles as well as in some ants, there is a need to explore the ecological implications of these associations and to investigate whether metabolically converged symbioses exhibit similarities in ecological function.

We investigated whether symbiosis contributes to desiccation resistance in *S. oryzae*, predicting that the elimination of the nutritional symbiont results in reduced fitness particularly under dry conditions. We exposed symbiotic and aposymbiotic beetles to long-term stressful or relaxed humidity conditions and compared population growth after 3 months. We found that symbiont absence severely constrained population growth, especially under desiccation stress. To understand the mechanisms underlying this effect, we compared beetle size, body water content, cuticular hydrocarbon profiles and the rate of water loss between symbiotic and aposymbiotic beetles. Results show that symbiotic beetles are bigger in size and have a higher body water content. While there were no significant differences in composition and quantity of cuticular hydrocarbons (CHCs) after long-term exposure to desiccation, symbiotic beetles lost water at a proportionally slower rate. We conclude that this desiccation resistance in symbiotic beetles is due to their thicker cuticle, which provides better prevention against evaporative water loss.

## Materials and methods

### Beetle rearing

Starter cultures of symbiotic and aposymbiotic *Sitophilus oryzae* were obtained from the “Laboratoire de Biologie Fonctionnelle, Insectes et interactions” (INSA Lyon, INRAE, France) in 2020. Symbionts had previously been eliminated using thermal stress as described in Nardon (1973). Briefly, adults aged between 1 and 3 weeks were kept for 28 days at 35°C and 90% relative humidity to obtain symbiont-free offspring beetles (aposymbiotic). Upon arrival in our laboratory, the population was expanded and maintained on organic wheat (Huber Mühle, Hohberg, Germany) in 1.8 L plastic containers with temperature and relative humidity set at 28°C and 60%, respectively. Prior to the start of the experiments, the symbiotic status of the beetles was confirmed following DNA extraction and qPCR protocols described in Vigneron et al. (2014).

### Chronic drought stress experiments

Beetles were exposed to long-term drought stress for 3 months. Here, two replicates of *N* = 30 beetles per treatment (symbiotic and aposymbiotic) were transferred to small plastic containers that were filled with 30 g of wheat. The beetles were then incubated at high (60% RH) or low humidity (47% RH). After 3 months of incubation, adult beetles were removed from the wheat and manually counted to determine the population growth (defined as the total accumulation of progenies) at this stage. The wheat was further incubated and adult offspring beetles that emerged from the wheat were frozen at −20°C in glass vials (1.4 mL) for later extraction of cuticular hydrocarbons and analysis via gas chromatography-mass spectrometry. As population growth was extremely constrained in aposymbiotic beetles at low humidity, all subsequent experimental comparisons of symbiotic and aposymbiotic beetles were conducted with the offspring beetles from the high humidity condition (that is, 60% RH).

### Symbiont impact on beetle size, body water content, and desiccation resistance

Gravimetric methods were used to determine symbiont impact on beetle body size and body water content using beetles that emerged from the 60% RH conditions. Here, symbiotic and aposymbiotic beetles (*N*_sym_ = 16; *N*_apo_ = 16) were freeze killed at −20°C and subsequently individually weighed to the nearest 0.001 mg using an electronic scale (Mettler Toledo, Giessen, Germany) to obtain the initial (wet) weight. Directly after being weighed, beetles were placed on small (3 cm × 3 cm) plastic weighing boats and dried overnight in an oven at 50°C; with a tray of silica gel beads to reduce humidity inside the oven. In addition, a subset of the dead beetles (*N*_sym_ = 10; *N*_apo_ = 9) were weighed at 2-h intervals over a period of 10 h to determine the rate of cuticular water loss. Beetles were weighed again after drying to obtain the dry weight. Body water content was determined for each individual as the difference between wet weight and dry weight. The proportional change in body water content was determined as water content divided by the wet weight and was used as a proxy for desiccation resistance.

### Chemical analysis of cuticular hydrocarbon profiles

To determine the physiological response of emerging beetles to desiccation stress, cuticular hydrocarbons (CHCs) were extracted from individuals symbiotic (*N*_sym_ = 33) and aposymbiotic (*N*_apo_ = 23) in 1.4 mL glass vials containing 20 μL of heptane and 1 μL (w/w 0.1%) of octadecane as internal standard (Alfa Aesar, Germany) for 20 min. The beetles were then removed, and the extracts analyzed with gas-chromatography-mass-spectrometry (GC-MS). GC-MS analyses were performed using an Agilent mass selective detector equipped with an inert extractor ion source (MSD5977B Inert Plus EI) coupled to an Agilent 8890 gas chromatograph. The GC was equipped with a HP-5 ms column (30 m × 0.25 mm ID; 0.25 μm df; Agilent, Santa Clara, CA, USA). The temperature of the split/splitless GC injector was set to 250°C, and the injector was operated in the splitless mode. The GC oven temperature was programmed as follows: 150°C for 1 min, heated with 5°C/min to 320°C and a final isothermal hold of 10 min. Helium was used as a carrier gas at a constant flow rate of 1 ml/min. Mass spectra were recorded with electron impact ionization with a mass range of m/z = 40–550 after a 5 min solvent delay until the end of the GC run time. The transfer line was operated at a temperature of 320°C, the ion source of 230°C and the quadrupol at 150°C. Compounds were identified in the MassHunter Quantitative Analysis software (V10.0, Agilent Technologies, Santa Clara, CA, USA) based on retention time and comparison with an external series of linear alkanes ranging from decane to tetracontane and manual interpretation of mass spectra following Nawrot et al. (1994). Quantification was performed with the Masshunter Quantitative Analysis software (V10.0 Agilent Technologies, Santa Clara, CA, USA). The total amount of CHCs were calculated for each beetle based on the internal octadecane standard.

### Statistical analysis

We determined the impact of symbiont status and humidity on population growth in the chronic drought stress experiment using a generalized linear mixed effects model (lme4 package, “glmer” command). The number of beetles counted after 3 months of exposure to desiccation stress was introduced as the response, while symbiont status, humidity and their interaction were fixed explanatory effects. Here, population replicate number was included in the model as a random effect. The effect of symbiont status on beetle weight and body water content were examined using Wilcoxon rank sum test. We fit a generalized linear mixed model to measure the influence of symbiont status on relative body water content over time (desiccation resistance), specifying individual ID as a random effect to account for repeated measures. We inspected the model residuals for temporal autocorrelation using ACF plots and refitted the model to account for temporal autocorrelation using the corAR1 correlation function (nlme package).

To examine the effects of symbiont status, treatment and their interaction on CHC profiles, we first performed multivariate analysis on centered log ratio transformed data (“clr” command) using PERMANOVAs based on Bray-Curtis dissimilarities with 9999 permutations (vegan package, “adonis” command). A matrix of all the relative CHC amounts was introduced into the analysis as the response, while symbiont status, treatment and their interaction were fixed explanatory effects. Thereafter, non-metric dimensional scaling (NMDS) ordination (“metaMDS” command) was performed using the relative abundances of the CHCs to visualize differences in CHC composition. The goodness of fit of the ordinations was evaluated by examining the stress value (acceptable when stress value < 0.2). We tested if there was a statistical difference between the groups on the NMDS ordination plot using ANOSIM tests (“anosim” command).

Finally, univariate features of CHCs that are known to be relevant for insect water balance were quantified and compared among treatments. To this end, for each beetle, we calculated the total amount of hydrocarbons (HCs), the proportion of unsaturated HCs (the sum of unsaturated HCs divided by the total amount of HCs) and the carbon chain length index (sum of the proportions of compounds multiplied by the number of carbons). GLMER models were then used to evaluate the influence of symbiont status, humidity and their interaction on each of the univariate parameters. Thus, total amount of HCs, proportion of unsaturated HCs or carbon chain length index were specified as response variables, while symbiont status, humidity and their interactions were fixed explanatory variables. The minimum adequate model was selected using backward model reduction. In all models, the replicate was specified as a random effect. All figures were done using the ggplot2 package.

## Results

We investigated whether symbiosis is beneficial to *S. oryzae* under desiccation stress, a condition that is ecologically relevant for grain pest beetles. For this, we examined symbiont influence on different beetle parameters, including population growth, weight, body water content, rate of water loss and cuticular hydrocarbons. The results of the different tests assessing symbiont influence on these parameters are given in Table 1.

**TABLE 1:** Effect of symbiont status and humidity on various beetle parameters.

Response	Test	Symbiont status	Humidity	Status* Humidity
Population growth	GLMER	*t* = −3.212 ***p* < 0.001**	*t* = 3.305 ***p* < *0.001***	*t* = 4.1471 ***p* < *0.001***
Wet weight (Ww)	Wilcoxon-rank sum test	*W* = *204 df* = *1* ***p* = *0.004412***	–	–
Dry weight (DW)	Wilcoxon-rank sum test	*W* = *208 df* = *1* ***p* = *0.0032723***		
Total body water content	Wilcoxon-rank sum test	*W* = *164 df* = *1* ***p* = *0.03864***	–	–
Relative body water content	Wilcoxon-rank sum test	*W* = *164 df* = *1* ***p* = *0.1807***	–	–
Water loss (desiccation resistance)	GLMER	*t* = 0.224 ***p* = 0.0463**	–	–
Total amount of CHCs	GLMER	*t* = 1.834 *p* = 0.7291	*t* = −1.8357 *p* = 0.2136	*t* = 0.8719 *p* = 0.2637
CHC carbon chain length	GLMER	*t* = −0.0955 *p* = 0.9239	*t* = −1.3873 *p* = 0.1653	*t* = 1.8739 *p* = 0.0609
CHCs: Proportion unsaturated	GLMER	*t* = 1.2354 *p* = 0.2167	*t* = −0.4369 *p* = 0.6621	*t* = 0.0695 *p* = 0.9445
CHCs	PERMANOVA	pseudo-f2 = 1.4320 *p* = 0.1856	pseudo-f2 = 0.8756 *p* = 0.5207	pseudo-f1 = 1.6623 *p* = 0.1224
CHCs	ANOSIM	*r* = 0.11 *p* = 0.0910	*r* = 0.03262 *p* = 0.1151	–

Significant *p*-values are given in bold.

*Denotes interaction effects.

### Symbiont impact on beetle fitness under chronic desiccation stress

**FIGURE 1:**
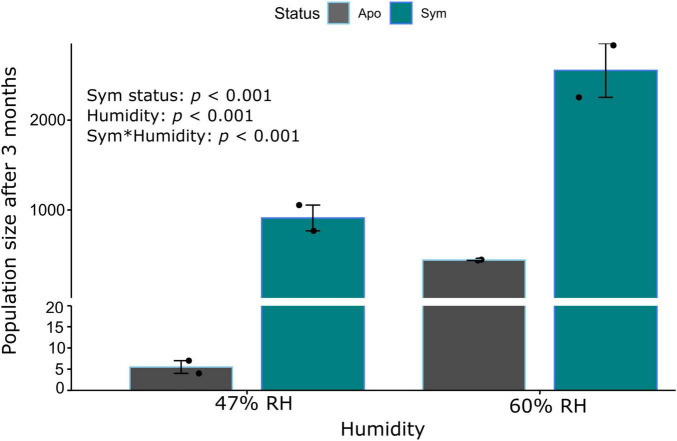
Symbiont impact on beetle population growth in *Sitophilus oryzae* under chronic desiccation stress. Barplot shows the mean ± SE of two replicate populations (black dots) of symbiotic (green bars) and aposymbiotic (gray bars) beetles after 3 months of exposure to low (47% RH) or high humidity (60% RH), respectively, starting from populations of 30 beetles. Population growth was significantly impacted by symbiont status, humidity and their interaction (GLMER, all *p* < 0.001).

To understand symbiont contribution to beetle fitness under dry conditions, we exposed beetles to drought stress over 3 months and compared their population growth to beetles kept under moister conditions. Population growth (the total accumulation of progenies) was significantly influenced by symbiont status (*p* < 0.001; GLMER; Table 1), humidity (*p* < 0.001; GLMER; Table 1), and their interaction (*p* < 0.001; GLMER; Table 1). In particular, the absence of symbionts under dry conditions severely constrained population growth (Figure 1). A 33-fold increase in population size was observed for symbiotic beetles over 3 months under dry stress conditions, while aposymbiotic beetles failed to increase in numbers in the same conditions (Figure 1).

### Symbiont impact on beetle size, body water content and desiccation resistance

**FIGURE 2:**
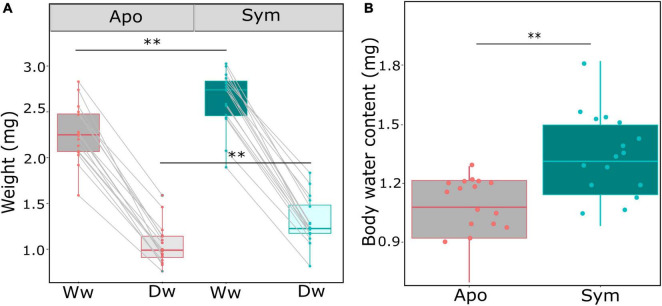
Symbiont impact on beetle weight and body water content. **(A)** Beetle weight is significantly impacted by symbiont status, and symbiotic beetles have a higher wet weight (Ww) and dry weight (Dw) than aposymbiotic beetles. **(B)** Total body content (wet weight–dry weight) differed significantly between symbiotic (green) and aposymbiotic (gray) beetles. Box plots show the median, 25th and 75th percentiles; the whiskers indicate the values within 1.5 times the interquartile range; the dots show individual aposymbiotic (red) and symbiotic (green) beetles. Asterisks denote significant differences by Wilcoxon rank sum tests: ^**^p < 0.01.

**FIGURE 3:**
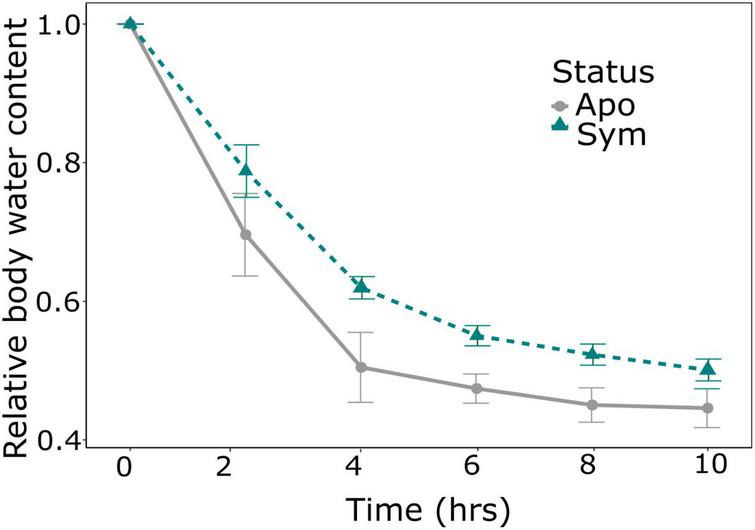
Impact of symbionts on water loss in *Sitophilus oryzae*. Symbiont status significantly influenced cumulative proportional water loss (change in weight) (*p* < 0.01, GLMER). Lines show the mean ± SE of symbiotic (green, *N* = 10) and aposymbiotic (gray, *N* = 9) beetles.

Due to surface area to volume considerations, an important aspect in insect water balance is body size, with smaller individuals predicted to lose water more rapidly than larger individuals. We thus compared the wet and dry weight of beetles of all treatments. Beetle weight was significantly influenced by symbiont status, with symbiotic beetles having ∼20% higher wet weight (*p* = 0.004; Wilcoxon rank sum test; Table 1; Figure 2A), and similarly increased dry weights (*p* = 0.003; Wilcoxon rank sum test; Table 1; Figure 2A). In addition, absolute body water content was significantly higher in symbiotic than in aposymbiotic beetles (*p* = 0.0039; Wilcoxon rank sum test; Table 1; Figure 2B), while relative body water content did not significantly differ between treatments (*p* = 0.1807; Wilcoxon rank sum test; Table 1; Figure 2B). To test whether these observed differences would influence desiccation resistance in *S. oryzae*, we measured the rate of cuticular water loss in both treatments over a 10-h period. Water loss was significantly influenced by symbiont status (*p* < 0.01; GLMER; Table 1), with symbiotic beetles losing water at a much slower rate than aposymbiotic beetles (Figure 3).

### Symbiont impact on cuticular hydrocarbons

**FIGURE 4:**
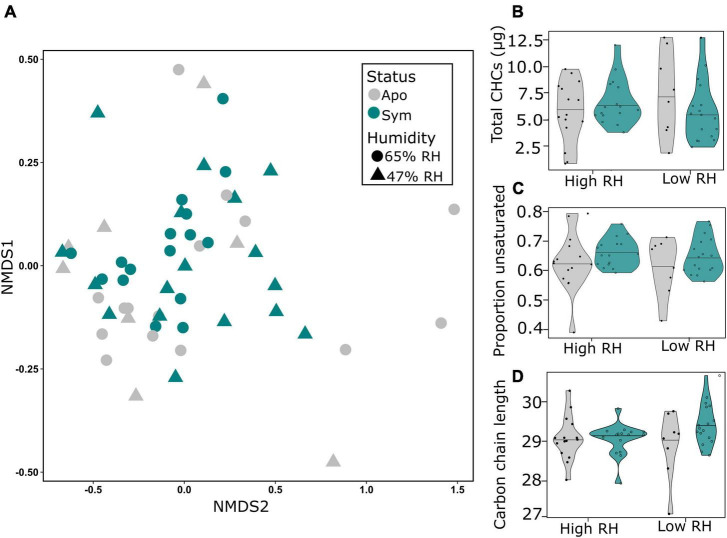
Symbionts do not impact cuticular hydrocarbons (CHCs) in *Sitophilus oryzae* under chronic desiccation stress. **(A)** Hydrocarbon profiles are visualized with non-metric dimensional scaling (NMDS) ordination based on Bray-Curtis dissimilarities. Each point represents the profile of a symbiotic (green) or aposymbiotic (gray) beetle. Different shapes represent different humidity regimes. Neither symbiont status nor humidity status significantly contributed to differences in profiles under chronic desiccation stress. Violin plots show **(B)** total CHCs, **(C)** proportion of unsaturated hydrocarbons and **(D)** carbon chain length index of symbiotic (green) and aposymbiotic (gray) beetles. Cuticular traits were not significantly affected by symbiont status, humidity or their interaction (*p* > 0.05; GLMER).

To determine whether symbiotic and aposymbiotic beetles make qualitative or quantitative adjustments to their epicuticle when confronted with dry stress, we extracted their cuticular hydrocarbons for chemical analysis by GC-MS. Multivariate analysis based on entire CHC profiles revealed that neither symbiont status nor humidity significantly affected the composition of CHCs (*p* > 0.05; PERMANOVA; Table 1; Figure 4A). Concordantly, a clear separation of the groups could not be observed by NMDS (Figure 4A), and further analysis of the matrix showed no significant differences between groups (*p* > 0.05 for both humidity and status; ANOSIM; Table 1). Similarly, when exposed to long-term drought, symbiotic and aposymbiotic beetles showed no significant differences in total abundance of CHCs, proportion of unsaturated hydrocarbons or carbon chain length index among treatments (all, *p* > 0.05; GLMER; Table 1; Figures 4B–D). However, a non-significant trend toward an interaction between symbiont status and humidity was observed for the average carbon chain length (*p* = 0.0609; GLMER, Table 1; Figure 4D).

## Discussion

The grain pest beetle *Sitophilus oryzae* is associated with the endosymbiont *Sodalis pierantonius*, which supplies the beetle with vitamins, essential amino acids and aromatic amino acids that are poorly represented in wheat grains (Vigneron et al., 2014). Additionally, the symbiont-provisioned aromatics play a pivotal role in enhancing both the melanization and thickness of the adult cuticle. However, the ecological benefit of the endosymbiosis had thus far not been demonstrated. In the present study, we show that endosymbionts confer a particularly strong fitness benefit to *S. oryzae* under desiccation stress, a condition that is characteristic of the dry storage facilities that the beetles infamously inhabit. Furthermore, we show that symbiotic beetles are more resistant to desiccation, losing water via their cuticle at a slower rate than aposymbiotic beetles.

Abiotic factors such as temperature and humidity can have a profound influence on insect fecundity, generation time, reproduction rate and mortality; and can thus impose severe selection pressures that determine species occurrences and abundances (Buxton, 1932; Khaliq et al., 2014; Dillon and Lozier, 2019). Concordantly, we found that low humidity significantly constrained population growth in *S. oryzae*, but this effect was exacerbated by endosymbiont absence. *S. pierantonius* endosymbiont encodes for multiple important vitamins and amino acids; and symbiont elimination decreases fecundity and increases larval developmental times (Heddi et al., 1999; Vigneron et al., 2014). This indicates that the nutritional supplements provided by the symbiont are important for host fitness. Despite this, aposymbiotic populations of *S. oryzae* can be maintained under lab conditions, albeit with lower population growth rates. Thus, the poor population growth observed in the aposymbiotic treatment under low humidity suggests that the absence of the symbionts under natural conditions (dehumidified storage facilities) would be highly detrimental, and that the symbionts provide an important fitness benefit, particularly under dry conditions. In agreement with this, no *S. oryzae* asymbiotic population was found in nature so far.

Insects can increase their resistance to desiccation through a combination of increasing initial body water content, reducing the rate of water loss or increasing tolerance to the amount of water that can be lost (Gibbs et al., 1998; Bazinet et al., 2010). We found that symbiotic beetles exhibit a larger body size and contain a significantly higher amount of water in the body. This was expected as variation in physiological traits, such as water and lipid content, is known to be strongly influenced by body size (Hadley et al., 1986). Higher levels of body water can increase survival odds when confronted with desiccation stress (Chown et al., 1999; Gibbs et al., 2004). However, mechanisms to reduce water loss can additionally serve to increase desiccation resistance. Concordantly, we observed that symbiotic beetles lose water via their cuticle at a proportionally slower rate than their aposymbiotic counterparts do. The caveat in our experiment is that we measured passive water loss since the beetles were dead, thus, any active mechanisms to reduce water loss are not accounted for. Nevertheless, we would still expect that a large portion of water is lost via the cuticle during desiccation stress in live insects. Studies have shown that cuticular transpiration is largely passive (Hadley et al., 1986) and accounts for >80% of the water loss during desiccation events (Gibbs et al., 1998, 2004; Fanning et al., 2019).

Permeability of the cuticle can be modified through structural changes such as its thickness but also by the incorporation of tyrosine-derived, hydrophobic compounds, or through the adjustment of the quantity and composition of epicuticular hydrocarbons (CHCs). All of these modifications can increase the hydrophobicity of cuticle and thereby reduce water loss (Stinziano et al., 2015; Noh et al., 2016; Ferveur et al., 2018). The enhanced thickness and melanization of the cuticle observed in symbiotic *S. oryzae* coincides with a massive increase in the density of the symbionts, which supply the precursors for the aromatic compounds that are in high demand for cuticle biosynthesis during metamorphosis (Vigneron et al., 2014). Thus, it is likely that the thicker cuticle of symbiotic beetles is responsible for the reduced rates of cuticular water loss observed in this treatment.

Insects can adjust the quantity and composition of CHCs to further waterproof the cuticle. Thus, we also analyzed the cuticular hydrocarbon profile of *S. oryzae* in an effort to understand the physiological mechanisms that may underpin the higher desiccation resistance observed in symbiotic beetles. Contrary to our expectations, neither symbiont status nor humidity significantly influenced the quantity or composition of CHCs. Interestingly, in another grain pest beetle that also harbors cuticle enhancing symbionts, namely *O. surinamensis*, experimentally symbiont-depleted beetles applied significantly more hydrocarbons to their cuticle upon short-term dry stress, likely as a way of compensating for their thinner cuticle (Engl et al., 2018). This indicates that when the structural integrity of the cuticle itself is compromised, beetles perceive desiccation stress more strongly and adjust CHC production to at least partially rescue water preservation by the cuticle. Why then do we not observe a similar effect in *S. oryzae*? One possible explanation might be that unlike *O. surinamensis*, females of *S. oryzae* oviposit inside grain kernels, wherein all larval stages and metamorphosis occur. Newly-enclosed adult beetles remain inside the grain for 3 days, during which time the cuticle starts developing before weevil’s emergence from the grain on the fourth day (Vigneron et al., 2014). It is also known that the infestation of grains by weevils increases the core temperature and humidity of grains (White et al., 2011). Thus, the within-grain “microhabitat” may provide sanctuary for the developmental stages that are most vulnerable to the harsh stressors of the external environment. It is also possible that there are adaptations beyond the upregulation of CHCs that become more important under long-term desiccation stress. This is supported by the observation that in *O. surinamensis*, chronic exposure to desiccation did not yield significantly different CHCs between treatments, even though symbiotic beetles showed higher population growths than aposymbiotic beetles. Moreover, *O. surinamensis* is in general capable of surviving and reproducing in much lower humidity than *S. oryzae* (Khan, 2009). Thus, there seems to be mechanisms and adaptations beyond the adjustment of the quantity of CHCs that make *O. surinamensis* specialized in surviving harsh conditions outside of grains. Concordantly, Gibbs et al. (1998) found that reduced rates of water loss in the desert fruit fly *Drosophila mojavensis* were more due to behavioral avoidance of high temperatures and desiccating conditions (that is, staying within rotting fruit) than they were to changes in cuticular lipids. This draws attention to the importance of “refuge” microhabitats for behavioral avoidance of unfavorable conditions. Thus, while *O. surinamensis* and *S. oryzae* occupy similar ecological niches and engage in functionally similar symbioses, they exhibit distinct differences in lifestyle, and likely employ different behavioral adaptations for dealing with dry stress.

It is noteworthy that while the total amount of CHCs and the proportion of unsaturated CHCs did not significantly differ between treatments, we observed a non-significant trend toward an interaction between symbiont status and humidity in the carbon chain length index. Specifically, symbiotic beetles tended to show an increase in average carbon chain length under desiccation stress. Longer chain hydrocarbons are thought to provide a better barrier against evaporation (Gibbs and Pomonis, 1995), and it is possible that this slight qualitative change might by facilitated by symbiont presence and confer some benefit to symbiotic beetles. Indeed, in many instances, it is changes in the composition of CHCs rather than the quantity of CHCs that drives variations in cuticular permeability, as has been demonstrated in tsetse flies, *Glossina pallidipes* (Jurenka et al., 2007) and in *Drosophila melanogaster* (Gibbs et al., 2004). Nevertheless, even in the absence of a significant change in CHCs, it is likely that the better performance of symbiotic vs. aposymbiotic *S. oryzae* under desiccation stress is due to symbiont-derived nutritional benefits that confer bigger body size, higher body water content, and a thicker cuticle.

By influencing the morphology and physiology of their insect hosts, symbiotic microbes play a pivotal role in influencing their hosts’ tolerance to abiotic challenges; and by extension, the niches that the hosts can occupy. There is a growing body of work highlighting the role played by microbial symbionts in rescuing their hosts from abiotic challenges such as temperature and drought stress (Dillon and Lozier, 2019; Lemoine et al., 2020). For instance, some secondary symbionts of aphids are heat tolerant and can expand the viable temperature range of their host, rescuing host survival during heat stress (Russell and Moran, 2006). Similarly, the cuticle enhancing symbionts of *O. surinamensis* were shown to enhance desiccation resistance and significantly improve fitness under dry conditions (Engl et al., 2018). We now show that the cuticle-enhancing symbionts of *S. oryzae* also confer a significant fitness benefit under desiccation stress, an ecologically relevant condition for grain pest beetles. Thus, we observe a similar ecological benefit in two beetles of different families with metabolically convergent symbioses. This likely extends to many other systems where microbe-mediated cuticle enhancement has been demonstrated, such as in the *Nardonella*-harboring weevils, but also in carpenter ants that harbor *Blochmannia* symbionts (Zientz et al., 2006; Kuriwada et al., 2010). Furthermore, symbiont contributions to cuticle biosynthesis, sclerotization, and melanization have been predicted based on symbiont genome information in other insect taxa, including beetles within the family Bostrichidae and in *Cardiocondyla* ants, where the symbionts have retained the pathways to synthesize tyrosine precursors in their highly eroded genomes (Klein et al., 2016; Kiefer et al., 2022). A similar ecological benefit can also be predicted for phylogenetically related symbionts such as the Flavobacteria of the Nosodendridae and Throscidae beetle families (Salem and Kaltenpoth, 2022). Thus, cuticle-enhancing symbionts occur across a broad diversity of insect taxa occupying different ecological niches, highlighting that insects can face similar ecological challenges in very different habitats and converge toward similar solutions.

## Data availability statement

Data are available from the data repository of the Max Planck Society (“Edmond”): https://doi.org/10.17617/3.D9Y0V3.

## Author contributions

MK and TE: conceptualization and supervision. MK, TE, and SK: methodology. TE and SK: software. SK: formal analysis, writing—original draft, and visualization. TE, MK, AH, and SK: writing—review and editing. MK: project administration and funding acquisition. All authors contributed to the article and approved the submitted version.
